# Effects of dietary live yeast supplementation on growth performance and biomarkers of metabolism and inflammation in heat-stressed and nutrient-restricted pigs^1^

**DOI:** 10.1093/tas/txab072

**Published:** 2021-05-27

**Authors:** Edith J Mayorga, Sara K Kvidera, Erin A Horst, Mohmmad Al-Qaisi, Carrie S McCarthy, Megan A Abeyta, Samantha Lei, Theodore H Elsasser, Stanislaw Kahl, Tadele G Kiros, Lance H Baumgard

**Affiliations:** 1 Department of Animal Science, Iowa State University, Ames, IA 50011, USA; 2 U.S. Department of Agriculture, Animal Biosciences and Biotechnology Laboratory, Beltsville, MD 20705, USA; 3 Phileo by Lesaffre, Milwaukee, WI 53214, USA

**Keywords:** growth performance, inflammation, live yeast, swine

## Abstract

Study objectives were to determine the effects of dietary live yeast (*Saccharomyces cerevisiae* strain CNCM I-4407; ActisafHR+; 0.25g/kg of feed; Phileo by Lesaffre, Milwaukee, WI) on growth performance and biomarkers of metabolism and inflammation in heat-stressed and nutrient-restricted pigs. Crossbred barrows (*n* = 96; 79 ± 1 kg body weight [**BW**]) were blocked by initial BW and randomly assigned to one of six dietary-environmental treatments: 1) thermoneutral (**TN**) and fed ad libitum the control diet (**TNCon**), 2) TN and fed ad libitum a yeast containing diet (**TNYeast**), 3) TN and pair-fed (**PF**) the control diet (**PFCon**), 4) TN and PF the yeast containing diet (**PFYeast**), 5) heat stress (**HS**) and fed ad libitum the control diet (**HSCon**), or 6) HS and fed ad libitum the yeast diet (**HSYeast**). Following 5 d of acclimation to individual pens, pigs were enrolled in two experimental periods (**P**). During P1 (7 d), pigs were housed in TN conditions (20 °C) and fed their respective dietary treatments ad libitum. During P2 (28 d), HSCon and HSYeast pigs were fed ad libitum and exposed to progressive cyclical HS (28–33 °C) while TN and PF pigs remained in TN conditions and were fed ad libitum or PF to their HSCon and HSYeast counterparts. Pigs exposed to HS had an overall increase in rectal temperature, skin temperature, and respiration rate compared to TN pigs (0.3 °C, 5.5 °C, and 23 breaths per minute, respectively; *P *< 0.01). During P2, average daily feed intake (**ADFI**) decreased in HS compared to TN pigs (30%; *P *< 0.01). Average daily gain and final BW decreased in HS relative to TN pigs (*P *< 0.01); however, no differences in feed efficiency (**G:F**) were observed between HS and TN treatments (*P *> 0.16). A tendency for decreased ADFI and increased G:F was observed in TNYeast relative to TNCon pigs (*P *< 0.10). Circulating insulin was similar between HS and TN pigs (*P *> 0.42). Triiodothyronine and thyroxine levels decreased in HS compared to TN treatments (~19% and 20%, respectively; *P *< 0.05). Plasma tumor necrosis factor-alpha (**TNF-α**) did not differ across treatments (*P *> 0.57) but tended to decrease in HSYeast relative to HSCon pigs (*P *= 0.09). In summary, dietary live yeast did not affect body temperature indices or growth performance and had minimal effects on biomarkers of metabolism; however, it tended to improve G:F under TN conditions and tended to reduce the proinflammatory mediator TNF-α during HS. Further research on the potential role of dietary live yeast in pigs during HS or nutrient restriction scenarios is warranted.

## INTRODUCTION

Heat stress (**HS**) negatively impacts all aspects of global agriculture and undermines animal welfare and productivity. In the swine industry, the costs of environmental hyperthermia are mainly explained by decreased and variable growth rates, altered carcass quality, poor sow performance, increased mortality and morbidity, and reduced facility efficiency (Baumgard and Rhoads, 2013; Ross et al., 2015). Many of these detrimental effects of HS can be attributed to HS-induced intestinal barrier dysfunction (Baumgard and Rhoads, 2013). During HS, blood is redistributed to the periphery in an effort to increase heat dissipation. This mechanism reduces blood flow to the gastrointestinal tract resulting in a spectrum of negative impacts on health and performance. At the least, the redirection in blood flow compromises nutrient absorption, while at the extreme, it can generate regional hypoxia and enterocyte damage (Lambert, 2009). Additionally, reduced nutrient intake, a hallmark of heat-stressed animals, has also been reported to compromise intestinal integrity (Pearce et al., 2013b; Kvidera et al., 2017c). Furthermore, evidence suggests that “stress” alone induces increased intestinal barrier permeability, a response likely mediated in part by intestinal mast cell activation (Santos et al., 2000; Moeser et al., 2007). Hyperpermeability of the intestinal epithelial barrier allows pathogen infiltration into circulation, stimulating a local and systemic inflammatory response (Mayorga et al., 2020). Once activated, the immune system utilizes a substantial amount of energy, and nutrients are partitioned away from productive purposes (i.e., growth, reproduction) to mount an immune response, negatively compromising animal health and productivity (Elsasser et al., 2008; Kvidera et al., 2017a, 2017b; Mayorga et al., 2020).

The use of dietary yeast has gained attention as it reportedly has positive effects on both ruminants and monogastrics (Broadway et al., 2015; Shurson, 2018; Burdick Sanchez et al., 2021). Particularly, dietary live yeast has been shown to improve growth rate, feed efficiency, milk production, and reproductive performance in various species (Di Giancamillo et al., 2007; de Ordanza et al., 2010; Nasiri et al., 2018). In relevance to gut health, dietary *Saccharomyces cerevisiae* improved multiple metrics in *Escherichia coli* challenged piglets (Trckova et al., 2014), effects seemingly mediated by improved intestinal permeability (Che et al., 2017). Furthermore, supplementing live yeast or yeast cell culture has been reported to be beneficial during HS by decreasing rectal temperature and improving feed intake, milk yield, and milk composition in dairy cows (Bruno et al., 2009; Moallem et al., 2009; Salvati et al., 2015). However, little is known regarding the role of dietary live yeast during HS in pigs. Therefore, it was of interest to evaluate whether live yeast supplementation can ameliorate the negative consequences of HS and nutrient restriction on metabolism and inflammation in a finishing-pig model.

## MATERIALS AND METHODS

### Animal, Diets, and Experimental Design

All procedures involving animals were approved by the Iowa State University Institutional Animal Care and Use Committee (#7-16-8316-S). Ninety-six barrows (79 ± 1 kg body weight [**BW**]), the progeny of PIC L42 dams × PIC L337 sires (PIC Inc., Hendersonville, TN), were utilized in a replicated (48 pigs per replicate, 1-week interval between replicates) experiment conducted at the Iowa State University Swine Nutrition Farm research facility (Ames, IA). Pigs were blocked by initial BW and randomly assigned to one of six dietary-environmental treatments: 1) thermoneutral (**TN**) conditions and ad libitum fed the control (**Con**) diet (**TNCon**; *n *= 12), 2) TN and ad libitum fed a live yeast supplemented diet (**TNYeast**; *n* = 12), 3) TN and pair-fed (**PF**) the Con diet (**PFCon**; *n *= 12), 4) TN and PF a live yeast supplemented diet (**PFYeast**; *n* = 12), 5) HS and ad libitum fed the Con diet (**HSCon**; *n* = 24), or 6) HS and ad libitum fed a live yeast supplemented diet (**HSYeast**; *n* = 24). Pigs were allocated to one of two environmentally controlled rooms. Each room had 24 crates (57 × 221 cm) where pigs were housed individually. Each crate was equipped with a stainless-steel feeder and a nipple drinker. Water was provided ad libitum during the entire experiment. One pig from the HSCon treatment was removed from the study due to health issues and data from it was not included in the analysis.

Two diets were formulated according to the following specifications: 1) a Con diet based on corn, soybean meal, and corn-distillers dried grains with solubles (**DDGS**) and 2) a diet supplemented with live yeast and based on corn, soybean meal, and corn DDGS (supplemented with live yeast at the rate of 0.25 g/kg of feed; *Saccharomyces cerevisiae* strain CNCM I-4407; ActisafHR+; Phileo by Lesaffre, Milwaukee, WI). Diets were processed in meal form at the Iowa State University Swine Nutrition Center Mill. Diets were formulated to meet or exceed the predicted requirements of finishing pigs (NRC, 2012) for energy, essential amino acids, protein, minerals, and vitamins (Table 1).

**Table 1.** Ingredient inclusion and chemical and nutritional characteristics of experimental diets (as-fed basis)

Item	Control	Live yeast
Ingredient, %		
Corn	64.10	64.10
Soybean meal	12.40	12.40
DDGS^1^	20.00	20.00
Soybean oil	1.25	1.25
l-Lysine HCL	0.25	0.25
Monocalcium phosphate	0.45	0.45
Limestone	1.00	1.00
NaCl	0.25	0.25
Vitamin Premix^2^	0.15	0.15
Trace Mineral Premix^3^	0.15	0.15
Live yeast^4^	-	0.025
Diet composition		
NE, kcal/kg	2476	2476
ME, kcal/kg	3321	3321
NE:ME	0.75	0.75
Crude protein, %	17.00	17.00
ADF, %	5.90	5.90
NDF, %	13.60	13.60
SID AA^5^, %		
Lys	0.73	0.73
Thr	0.47	0.47
Met	0.25	0.25
Trp	0.13	0.13
Ca, %	0.52	0.52
Total P	0.50	0.50
STTD^6^ P	0.28	0.28

^1^Corn distillers dried grains with solubles.

^2^Vitamin premix provided the following (per kg diet): 4900 IU of vitamin A, 560 IU of vitamin D_3_, 40 IU of vitamin E, 2.4 mg of menadione (to provide vitamin K), 39 μg of vitamin B_12_, 9 mg of riboflavin, 22 mg of d-pantothenic acid, and 45 mg of niacin.

^3^Mineral premix provided the following (per kg diet): 165 mg of Fe (ferrous sulfate), 165 mg of Zn (zinc sulfate), 39 mg of Mn (manganese sulfate), 2 mg of Cu (copper sulfate), 0.3 mg of I (calcium iodate), and 0.3 mg of Se (sodium selenite).

^4^ActiSafHR+ (*Saccharomyces cerevisiae* CNCM I-4407, Phileo by Lesaffre, Milwaukee, WI).

^5^Standardized ileal digestibility.

^6^Standardized total tract digestibility.

**Figure 1.**
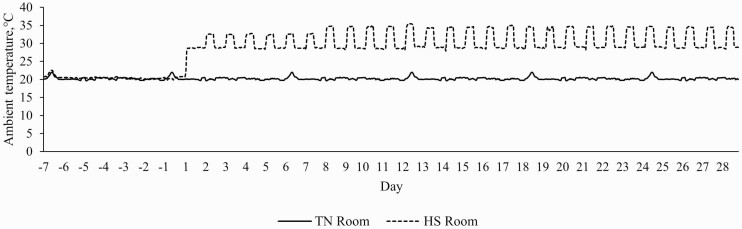
Ambient temperature (°C) in the thermoneutral (**TN**; 20.37 ± 0.02 °C, 57.15 ± 0.30% relative humidity) and heat stress (**HS**; 28–33 °C, 37.79 ± 0.20% relative humidity) rooms during period 1 (d -7 to d -1) and period 2 (d 1 to d 28).

The study consisted of two experimental periods (**P**): P1 and P2. Prior to the start of the study, pigs were moved to individual crates, allowed to acclimate for 5 d in TN conditions (20.37 ± 0.02 °C, 56.18 ± 0.45% relative humidity) with a 12 h:12 h light-dark cycle, and fed the Con diet ad libitum. During P1 (7 d), pigs were fed their respective dietary treatments ad libitum and kept in TN conditions for collection of baseline body temperature indices and production parameters. During P2 (28 d), HSCon and HSYeast animals were fed ad libitum and exposed to progressive cyclical HS conditions with temperatures ranging from 28 °C to 33 °C (d 1: 28.06 ± 0.57 °C from 0800 to 1800 h and 28.77 ± 0.03 °C from 1800 to 0800; d 2 to d 7: 31.69 ± 0.17 °C from 0800 to 1800 h and 28.69 ± 0.01 °C from 1800 to 0800 h; d 8 to d 28: 33.25 ± 0.14 °C from 0800 to 1800 h, and 28.81 ± 0.01 °C from 1800 to 0800; Figure 1). The room was heated using a propane forced air furnace (Model AW250, L.B. White Co. Inc., Onalaska, WI) with a maximum and minimum input of 250,000 and 160,000 BTU, respectively, and blower outlet of 1.83 m. The heaters were located in one corner of the room and fans evenly distributed the air. Room temperature and relative humidity were monitored and recorded every 5 min by a data logger (Lascar EL-USB-2-LCD; Erie, PA). One data logger was placed in each quadrant of each room (eight data loggers in total) and data were condensed into hourly averages by room.

During P2, pigs assigned to the TNCon and TNYeast treatments remained in TN conditions and were fed ad libitum. Pigs in PFCon and PFYeast treatments also remained in TN conditions, but were PF to their respective HS counterparts to eliminate the confounding effects of dissimilar nutrient intake. In brief, average daily feed intake (**ADFI**) during P1 was averaged for each pig and used as a baseline. During P2, the decrease in ADFI in HS pigs was calculated every day as a percentage of ADFI reduction relative to P1. The percentage of ADFI reduction was averaged for all pigs in the HS treatments per day of HS exposure and applied individually to the baseline of each pig in the PF treatments. The daily amount of feed provided was divided into two equal portions during P2 (~0800 and 1800 h) to minimize large metabolic changes associated with gorging. In order to accomplish pair-feeding, pigs assigned to the PF treatments remained 2 calendar days behind TN and HS treatments throughout the experimental period. This pair-feeding technique has been extensively utilized (Pearce et al., 2013a; 2015; Sanz Fernandez et al., 2015a, b).

### Body Temperature Measurements

During both P1 and P2, body temperature indices (rectal temperature [**T**_**R**_], skin temperature [**T**_**S**_], and respiration rate [**RR**]) were obtained twice daily (~0700 and 1800 h). Rectal temperature was measured using a calibrated electronic thermometer (SureTemp Plus 690, accuracy: ±0.1 °C; WelchAllyn; Skaneateles Falls, NY). Skin temperature was measured at the rump using a calibrated infrared thermometer (IRT207: The Heat Seeker 8:1 Mid-Range Infrared Thermometer, accuracy: ±2 °C; General Tools; New York, NY) ~12 cm from the skin. Respiration rate was determined by counting flank movements in a 15 s interval and multiplied by 4 to obtain breaths per minute (**bpm**). All indices recorded were condensed into AM, PM, and daily averages.

### Production Parameters

Average daily feed intake was measured during P1 and P2 as feed disappearance. Body weights were obtained at the beginning of acclimation, at the beginning and at the end of P1, and on d 7, 14, 21, and 28 of P2. Average daily gain (**ADG**) and feed efficiency (**G:F**) were calculated by week during P1 and P2.

### Blood Sampling

Blood samples were obtained by jugular venipuncture (plasma, K_2_EDTA tube; serum, plastic tube; BD vacutainers; Franklin Lakes, NJ) on d 7 of P1 and d 7 and 28 of P2 from nonfasted snared pigs. Serum and plasma samples were harvested by centrifugation at 1500 × *g* for 15 min at 4 °C, aliquoted into 2 mL microcentrifuge tubes, and stored at –20 °C until analysis. Circulating glucose, insulin, NEFA, blood urea nitrogen (**BUN**), cortisol, and tumor necrosis factor alpha (**TNF-α**) concentrations were measured using commercially available kits as previously described (glucose, Wako Chemicals USA, Inc, Richmond, VA; insulin, Mercodia Porcine Insulin ELISA; Mercodia AB, Uppsala, Sweden; NEFA, Wako Chemicals USA, Inc., Richmond, VA; BUN, Teco Diagnostics, Anaheim, CA; cortisol, Enzo Life sciences Inc., Farmingdale, NY; TNF-α, R&D Systems, Inc., Minneapolis, MN). The intra- and inter-assay coefficients of variation for glucose, insulin, NEFA, BUN, cortisol, and TNF-α were 4.1% and 9.1%, 5.9% and 5.5%, 3.9% and 11.5%, 3.9% and 11.8%, 5.9% and 24.4%, and 3.3% and 2.4%, respectively. Total plasma triiodothyronine (**T**_**3**_) and thyroxine (**T**_**4**_) concentrations were evaluated using commercially available solid phase RIA kits (MP Biomedicals, LLC; Irvine, CA) according to the manufacturer’s instructions; the assay kits were validated (recovery and linearity of diluted samples) for use with porcine plasma samples as previously described (Kahl et al., 2000).

### Statistical Analysis

Data were statistically analyzed using SAS version 9.4 (SAS Institute Inc., Cary, NC). Daily measurements (i.e., ADFI and body temperature indices) were condensed into weekly means. Body temperature indices, production parameters, and blood metabolites were analyzed using the MIXED procedure of SAS with an autoregressive covariance structure and week of the experiment as the repeated effect. The model included treatment, week, treatment by week interaction, block, and replicate as fixed effects; BW during P1 (i.e., BW taken on d 1 of P1 before feeding respective dietary treatments) was included in the model as a covariate. Final BW was analyzed using PROC MIXED with a diagonal covariance structure and the initial BW as a covariate. Pre-planned contrasts were assessed to evaluate the effects of the environmental treatment (i.e., TN vs. PF, TN vs. HS, and PF vs. HS) and the effects of the diet (i.e., Con vs. live yeast). Results are reported as least square means and considered significant if *P *≤ 0.05 and a tendency if 0.05 < *P* ≤ 0.10.

## RESULTS

### Body Temperature Indices

**Figure 2.**
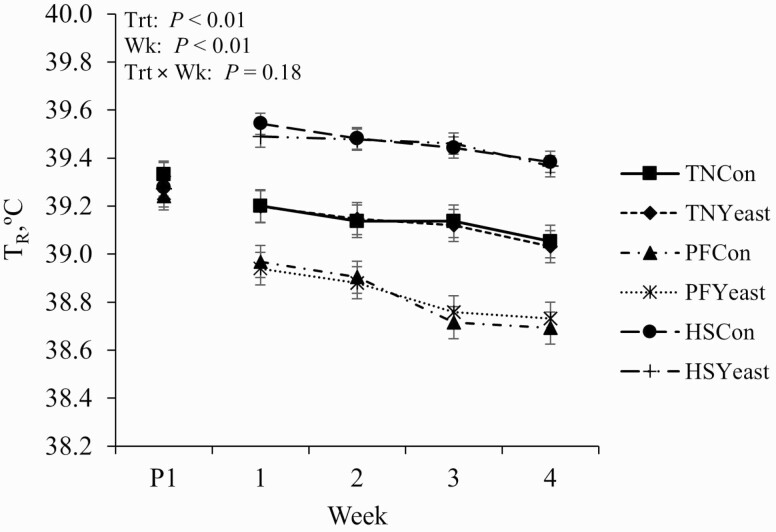
Effects of live yeast supplementation on weekly rectal temperature (**T**_**R**_) during period 2. P1 represents the average of rectal temperature obtained during the 7 d of period 1. Treatments: TNCon = thermoneutral (**TN**) and ad libitum fed the control diet, TNYeast = TN and ad libitum fed the yeast diet, PFCon = TN and pair-fed the control diet, PFYeast = TN and pair-fed the yeast diet; HSCon = heat stress and ad libitum fed the control diet, and HSYeast = heat stress and ad libitum fed the yeast diet. Data are represented as least squares means ± standard error of the mean and considered significant if *P* ≤ 0.05 and a tendency if 0.05 < *P *≤ 0.10.

Pigs exposed to HS had an overall increase in T_R_ relative to TN and PF treatments (~0.3 °C and 0.6 °C, respectively; *P* < 0.01; Table 2). Skin temperature was also increased in HS pigs relative to both TN and PF pigs (5.5 °C and 7.3 °C, respectively; *P* < 0.01; Table 2). Additionally, RR increased in HS animals relative to TN and PF pigs (~23 and 30 bpm, respectively; *P* < 0.01; Table 2). Furthermore, PF pigs had decreased T_R_, T_S_, and RR relative to their TN counterparts (~0.3 °C, 1.8 °C, and 7 bpm, respectively; *P* ≤ 0.01; Figure 2).

**Table 2.** Effects of live yeast supplementation on body temperature indices in thermoneutral, nutrient-restricted, and heat-stressed pigs

Parameter	Treatment^1^						SEM	*P*-value			Contrasts^5^			
	TN Con	TN Yeast	PF Con	PF Yeast	HS Con	HS Yeast		Trt^2^	Wk^3^	Trt × Wk^4^	TN vs. PF	TN vs. HS	PF vs. HS	Con vs. Yeast
T_R_^6^, C°	39.13^a^	39.12^a^	38.82^b^	38.83^b^	39.46^c^	39.45^c^	0.05	<0.01	<0.01	0.18	<0.01	<0.01	<0.01	0.90
0700 h	39.05^c^	39.03^c^	38.72^a^	38.75^a^	39.05^c^	39.12^c^	0.04	<0.01	<0.01	<0.01	<0.01	0.28	<0.01	0.38
1800 h	39.22^a^	39.21^a^	38.92^b^	38.91^b^	39.87^c^	39.77^c^	0.07	<0.01	<0.01	0.25	<0.01	<0.01	<0.01	0.43
T_S_^7^, C°	32.51^a^	33.02^a^	31.00^b^	31.01^b^	38.23^c^	38.32^c^	0.18	<0.01	<0.01	<0.01	<0.01	<0.01	<0.01	0.15
0700 h	31.82^b^	32.52^a^	30.21^d^	30.46^d^	37.14^c^	37.25^c^	0.20	<0.01	<0.01	<0.01	<0.01	<0.01	<0.01	0.02
1800 h	33.20^a^	33.51^a^	31.79^b^	31.56^b^	39.32^c^	39.39^c^	0.17	<0.01	<0.01	<0.01	<0.01	<0.01	<0.01	0.72
RR^8^, bpm	49^a^	48^a^	43^ab^	41^b^	71^c^	72^c^	2	<0.01	<0.01	0.24	0.01	<0.01	<0.01	0.70
0700 h	44^a^	42^ab^	38^ab^	37^b^	58^c^	60^c^	2	<0.01	<0.01	0.03	0.02	<0.01	<0.01	0.99
1800 h	53^a^	53^a^	48^ab^	45^b^	84^c^	83^c^	3	<0.01	<0.01	0.01	0.03	<0.01	<0.01	0.54

^1^TNCon = thermoneutral control; TNYeast = thermoneutral yeast; PFCon = pair-fed control; PFYeast = pair-fed yeast; HSCon = heat stress control; HSYeast = heat stress yeast.

^2^Treatment.

^3^Week (4 wk).

^4^Treatment by week interaction.

^5^TN = TNCon+TNYeast; PF = PFCon+PFYeast; HS = HSCon+HSYeast; Con = TNCon+PFCon+HSCon; Yeast = TNYeast+PFYeast+HSYeast.

^6^Rectal temperature averaged by day.

^7^Skin temperature averaged by day.

^8^Respiration rate averaged by day.

^a–d^Means within a row with different superscripts significantly differ (*P* ≤ 0.05).

Overall (irrespective of environment), there were no differences in T_R_ or RR between Con and live yeast-fed pigs (*P* > 0.10); however, T_S_ (0700 h) was increased in live yeast- relative to Con-fed pigs (~0.4 °C; *P* = 0.02; Table 2).

### Production Parameters

**Figure 3.**
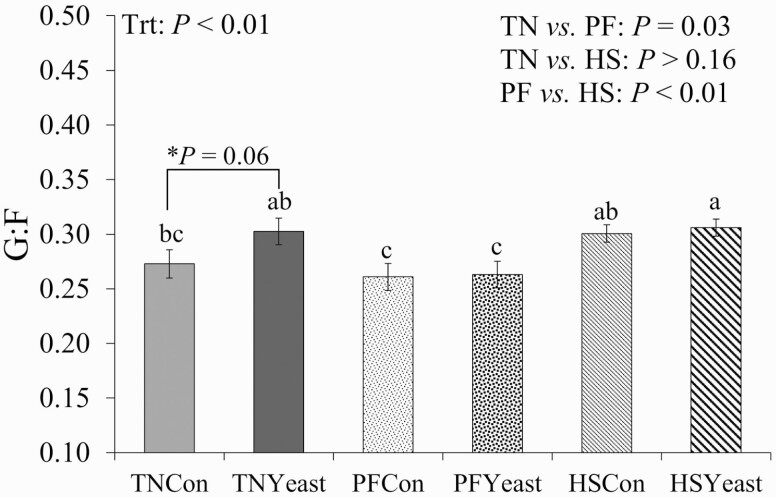
Effects of live yeast supplementation on feed efficiency (**G:F**) during period 2. Treatments: TNCon = thermoneutral (**TN**) and ad libitum fed the control diet, TNYeast = TN and ad libitum fed the yeast diet, PFCon = TN and pair-fed the control diet, PFYeast = TN and pair-fed the yeast diet; HSCon = heat stress and ad libitum fed the control diet, and HSYeast = heat stress and ad libitum fed the yeast diet. ^a–c^Values with different superscripts denote differences (*P *< 0.05) between treatments. ^*^Post-hoc analysis denoting a tendency (*P* < 0.10) for the contrast TNCon vs. TNYeast. Data are represented as least squares means ± standard error of the mean and considered significant if *P* ≤ 0.05 and a tendency if 0.05 < *P* ≤ 0.10.

As expected, ADFI was decreased during P2 in HS pigs relative to TN treatments (~1.1 kg/d; 30%; *P *< 0.01; Table 3). Similarly, ADG was reduced in pigs exposed to HS compared to TN controls (~0.3 kg/d; 26%; *P *< 0.01; Table 3). Additionally, there was a treatment by week interaction (*P* < 0.01) as both HS treatments had decreased (58%) ADG compared to PF controls during week 1, but similar rates of growth during weeks 2–4 (data not shown). Final BW was also decreased in HS treatments (~8.1 kg; 6%; *P *< 0.01; Table 3) relative to TN treatments. No differences were observed on G:F between HS and TN pigs during P2 (*P* > 0.16; Figure 3). By experimental design, PF pigs had a similar pattern and extent in ADFI reduction, and their final BW was similar to their HS counterparts (*P* > 0.10). Additionally, ADG and G:F variables were decreased in PF when compared to both TN and HS pigs (*P* < 0.05). Although no differences were observed on production parameters when comparing Con vs. live yeast-fed pigs (*P* > 0.10), TNYeast pigs had a tendency for decreased ADFI (~0.3 kg/d; *P* = 0.07; driven largely by differences in weeks 1 and 2; treatment by week interaction: *P* < 0.01; data not shown) and a tendency for increased G:F (~11%; *P* = 0.06) when compared to their TNCon counterparts (Figure 3).

**Table 3.** Effects of live yeast supplementation on growth performance in thermoneutral, nutrient-restricted, and heat-stressed pigs

	Treatment^1^						SEM	*P*-value			Contrasts^5^			
Parameter	TN Con	TN Yeast	PF Con	PF Yeast	HS Con	HS Yeast		Trt^2^	Wk^3^	Trt × Wk^4^	TN vs. PF	TN vs. HS	PF vs. HS	Con vs. Yeast
ADFI, kg	3.81^a^	3.55^a^	2.71^b^	2.62^b^	2.56^b^	2.60^b^	0.10	<0.01	0.57	<0.01	<0.01	<0.01	0.41	0.18
ADG. kg	1.04^a^	1.08^a^	0.71^bc^	0.69^c^	0.77^bc^	0.79^b^	0.03	<0.01	<0.01	<0.01	<0.01	<0.01	0.02	0.63
IBW^6^, kg	78.1	78.6	79.1	79.6	79.9	79.5	1.0	0.76	-	-	0.30	0.17	0.73	0.82
FBW^7^, kg	123^a^	124^a^	113^b^	113^b^	115^b^	116^b^	1	<0.01	-	-	<0.01	<0.01	0.12	0.47

^1^TNCon = thermoneutral control; TNYeast = thermoneutral yeast; PFCon = pair-fed control; PFYeast = pair-fed yeast; HSCon = heat-stress control; HSYeast = heat-stress yeast.

^2^Treatment.

^3^Week (4 wk).

^4^Treatment by week interactions.

^5^TN = TNCon+TNYeast; PF = PFCon+PFYeast; HS = HSCon+HSYeast; Con = TNCon+PFCon+HSCon; Yeast = TNYeast+PFYeast+HSYeast.

^6^Initial body weight.

^7^Final body weight.

^a–c^Means with different superscripts significantly differ (*P* ≤ 0.05).

### Blood Parameters

During P2, circulating glucose was similar across experimental treatments (*P* > 0.11; Table 4); however, glucose tended to be decreased and was decreased in HS relative to TN and PF pigs (~6%, *P* = 0.10 and 10%, *P* = 0.01, respectively). No differences in blood glucose levels were observed between Con and live yeast-fed pigs (*P *> 0.77).

**Table 4.** Effects of live yeast supplementation on blood metabolites and inflammation in thermoneutral, nutrient-restricted, and heat-stressed pigs

Parameter	Treatment^1^						SEM	*P*-value			Contrasts^5^			
	TN Con	TN Yeast	PF Con	PF Yeast	HS Con	HS Yeast		Trt^2^	Wk^3^	Trt × Wk^4^	TN vs. PF	TN vs. HS	PF vs. HS	Con vs. Yeast
Glucose, mg/dl	91.3	93.7	98.0	94.5	84.9	88.3	3.5	0.11	0.16	0.46	0.32	0.10	0.01	0.77
Insulin, μg/l	0.12^xy^	0.12^x^	0.06^y^	0.06^y^	0.12^x^	0.09^xy^	0.02	0.09	0.30	0.13	0.01	0.42	0.03	0.54
NEFA^6^, μE/l	107^a^	106^a^	167^b^	172^b^	88^a^	78^a^	10	<0.01	0.05	0.24	<0.01	0.07	<0.01	0.87
BUN^7^, mg/dl	11.5	10.3	9.1	11.4	10.2	10.5	0.79	0.32	0.01	0.05	0.45	0.41	0.95	0.44
Cortisol, ng/ml	13.0	19.3	14.6	23.8	19.2	16.6	4.7	0.70	0.24	<0.01	0.57	0.71	0.78	0.28
T_3_^8^, ng/ml	0.45	0.51	0.45	0.43	0.38	0.40	0.04	0.27	<0.01	0.99	0.39	0.03	0.24	0.45
T_4_^9^, ng/ml	36.0^b^	39.7^b^	36.8^b^	34.3^ab^	29.6^a^	31.0^a^	2.1	0.01	<0.01	0.18	0.32	<0.01	0.02	0.63
T_3_:T_4_^10^, ×10^2^	1.58	1.58	1.50	1.55	1.58	1.54	0.17	0.99	0.29	0.41	0.78	0.90	0.85	0.98

^1^TNCon = thermoneutral control; TNYeast = thermoneutral yeast; PFCon = pair-fed control; PFYeast = pair-fed yeast; HSCon = heat stress control; HSYeast = heat stress yeast.

^2^Treatment.

^3^Week (4 wk).

^4^Treatment by week interactions.

^5^TN = TNCon+TNYeast; PF = PFCon+PFYeast; HS = HSCon+HSYeast; Con = TNCon+PFCon+HSCon; Yeast = TNYeast+PFYeast+HSYeast.

^6^Non-esterified fatty acids.

^7^Blood urea nitrogen.

^8^Triiodothyronine.

^9^Thyroxine.

^10^Triiodothyronine to thyroxine ratio (molar ratio).

^a–b^Means with different superscripts denote an overall treatment difference (*P* ≤ 0.05).

^x–y^Means with different superscripts denote an overall treatment tendency (0.05 < *P* ≤ 0.10).

Despite marked differences in ADFI during P2, insulin levels did not differ between TN and HS treatments (*P *> 0.42; Table 4). However, PF pigs had decreased plasma insulin relative to both TN and HS pigs (~50% and 43%, respectively; *P* ≤ 0.03). When comparing Con vs. live yeast-fed pigs, no differences were observed in circulating insulin (*P* > 0.54).

During P2, plasma NEFA levels tended to be decreased in HS pigs relative to their TN counterparts (~22%; *P* = 0.07; Table 4). Contrarily, circulating NEFA was increased in PF pigs compared to both TN and HS treatments (~59% and 104%, respectively; *P* < 0.01). No differences were observed in circulating NEFA between Con and live yeast-fed pigs (*P *> 0.87). No treatment differences were observed in BUN during P2 across treatments (*P* > 0.32; Table 4) or between Con and live yeast-fed pigs (*P* > 0.44). During P2, circulating cortisol did not differ among treatments (*P* > 0.70; Table 4). Additionally, no differences were detected between Con and live yeast-fed pigs (*P* > 0.28).

No treatment effects were observed on circulating T_3_ during P2 (*P* > 0.27; Table 4). However, when comparing across environmental treatments, T_3_ was decreased in HS relative to TN pigs (~19%; *P* = 0.03). Additionally, no differences were observed on T_3_ when comparing Con vs. live yeast-fed pigs (*P* > 0.45). Circulating T_4_ levels were decreased during P2 in HS treatments relative to both TN and PF pigs (~20% and 15%, respectively; *P *≤ 0.02; Table 4). Overall, no differences were observed in T_4_ during P2 between Con and live yeast-fed pigs (*P* > 0.63). The T_3_:T_4_ did not differ across treatments during P2 (*P* > 0.99). Likewise, no differences were observed on the T_3_:T_4_ between Con and live yeast-fed pigs (*P* > 0.98; Table 4).

**Figure 4.**
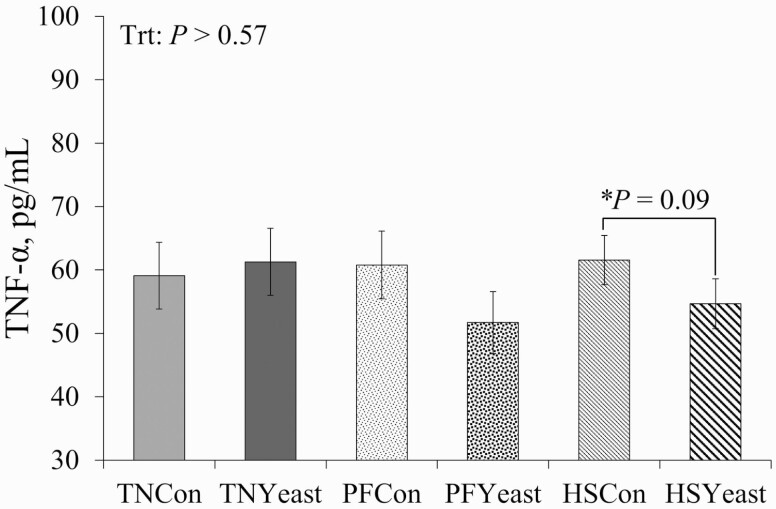
Effects of live yeast supplementation on circulating tumor necrosis factor-alpha (**TNF-α**) during period 2. Treatments: TNCon = thermoneutral (**TN**) and ad libitum fed the control diet, TNYeast = TN and ad libitum fed the yeast diet, PFCon = TN and pair-fed the control diet, PFYeast = TN and pair-fed the yeast diet; HSCon = heat stress and ad libitum fed the control diet, and HSYeast = heat stress and ad libitum fed the yeast diet. ^*^Post hoc analysis denoting a tendency (*P* < 0.10) for the comparison between HSCon and HSYeast treatments. Data are represented as least squares means ± standard error of the mean and considered significant if *P* ≤ 0.05 and a tendency if 0.05 < *P *≤ 0.10.

Circulating TNF-α levels were similar across experimental treatments during P2 (*P* > 0.57; Figure 4). However, a treatment by week interaction was observed for TNF-α as it progressively decreased in TN and HS pigs during P2, while it increased over time in PF pigs (*P* = 0.05; data not shown). Although no differences on plasma TNF-α were detected between Con- and live yeast-fed pigs (*P *> 0.25), a post hoc analysis revealed a tendency for decreased TNF-α in HSYeast when compared to their HSCon counterparts (14%; *P* = 0.09; Figure 4).

## DISCUSSION

Heat stress represents one of the costliest issues in animal agriculture as it compromises almost every key performance indicator. While advances in management practices (i.e., cooling systems) have partially ameliorated the negative consequences of HS, animal productivity remains decreased during the warm summer months. Due to their low ability to dissipate heat, pigs are especially sensitive to HS; therefore, they rely on reducing metabolic heat production by decreasing ADFI, physical activity, and skeletal muscle accretion (Renaudeau et al., 2012; Mayorga et al., 2020). Furthermore, animals alter their carbohydrate, lipid, and protein metabolism (independently of ADFI and energy balance) in an effort to maintain euthermia (Baumgard and Rhoads, 2013). Altered fuel supply and use by different tissues is a hallmark characteristic of HS that ultimately results in decreased productivity.

Although mechanisms by which HS alters nutrient partitioning are not entirely clear, a possible explanation relies on the detrimental effects of HS on intestinal integrity. Decreased intestinal barrier function allows for the translocation of microbial (i.e., lipopolysaccharide [**LPS**]) and dietary antigens into portal and systemic circulation, stimulating an inflammatory response (Lambert, 2009; Baumgard and Rhoads, 2013). Once activated, the immune system requires substantial amounts of energy, and nutrients are shifted towards the immune system and away from growth and other productive purposes (Kvidera et al., 2017a, 2017b). Therefore, dietary interventions aimed at improving gut health and the inflammatory response during HS or nutrient restriction scenarios are of particular interest. Dietary yeast is thought to improve animal performance by maintaining a beneficial intestinal environment, preventing pathogenic bacteria from binding to enterocytes, improving intestinal permeability, and by modulating immune function (Vohra et al., 2016; Che et al.., 2017; Shurson et al., 2018, Burdick Sanchez et al., 2021). Therefore, we hypothesized that supplementing live yeast would ameliorate some of the adverse effects of HS and nutrient restriction on gut health, which would translate into improved growth performance metrics.

Exposing pigs to an environmental heat load caused marked changes in all body temperature measurements. Increased T_R_, T_S_, and RR were observed in HS pigs compared to TN animals, indicating the HS protocol was successfully implemented. In contrast, PF pigs had decreased T_R_, T_S_, and RR relative to the TN pigs, as previously reported (Pearce et al., 2013a; Seibert et al., 2018; Mayorga et al., 2019); presumably, an affect associated with the reduced thermal effect of feeding. Although dietary live yeast supplementation had no effects on T_R_ or RR during HS, a slightly increased T_S_ (+0.4 °C; 0700 h) was observed in live yeast-fed pigs when compared to their Con counterparts (regardless of environmental treatment). Explanations for this are not clear, especially as pigs in both treatments had similar feed intake and growth rate (two key contributors to metabolic heat production). Regardless, whether the small increase in T_S_ in live yeast-fed pigs is biologically relevant remains unknown but of interest.

As expected, HS exposure decreased ADFI (~30%), ADG (~26%), and final BW (~7%) relative to a TN environment; by experimental design, PF pigs followed this same pattern of reduced nutrient intake and compromised growth performance. The extent and magnitude of reduced growth performance observed in the current study are comparable to previous HS trials (Johnson et al., 2015; Mayorga et al., 2019). Decreased ADFI during a thermal load has been extensively reported within the literature (as reviewed by Nyachoti et al., 2004; Mayorga et al., 2020) and represents an effort to minimize metabolic heat production. Reduced ADFI obviously contributes a large portion to the overall decline in growth performance; however, changes in metabolism observed between HS and PF animals, despite both being under the same plane of nutrition, suggest HS has direct and indirect (via reduced ADFI) effects on animal productivity (Baumgard and Rhoads, 2013).

Feed efficiency was similar between TN and HS pigs but was increased in HS relative to PF pigs (0.31 vs. 0.26 kg gain/kg feed, respectively). This is of particular relevance as the literature suggests HS compromises G:F, presumably by increasing maintenance requirements (Renaudeau et al., 2012). Increased energy costs during HS are associated with energy expenditure for heat dissipation processes (i.e., panting and sweating) and because the Van’t Hoff–Arrhenius equation predicts greater chemical reaction rates with increasing temperatures (Kleiber, 1961; Fuquay, 1981). In fact, increased maintenance costs during HS have been reported in multiple species, including rodents, poultry, and cattle (Collins et al., 1980; Beede and Collier, 1986; Yahav, 2007). Although previous observations on the effects on HS on G:F have been small and inconsistent (Lefaucheur et al., 1991; Collin et al., 2001; Kerr et al., 2003; Renaudeau et al., 2008, 2011), improved G:F during HS in the current study may be associated to decreased circulating thyroid hormones (Johnson et al., 2015; Sanz Fernandez et al., 2015a; further discussed below), reduced splanchnic mass (Johnson et al., 2015), as well as reduced ADFI and physical activity observed in HS pigs; suggesting energy expenditure may actually decrease during a heat load (Collin et al., 2001; Brown-Brandl et al., 2004). Thus, differences in G:F observed between HS and PF pigs despite both being under the same plane of nutrition, warrant further investigation. Likely, changes in lipid metabolism during HS allow for increased carcass lipid retention and partially explain the differences in ADG observed between HS and PF pigs (Baumgard and Rhoads, 2013).

Contrary to our hypothesis, no differences in growth performance were observed due to live yeast supplementation during HS. However, a tendency for increased G:F during TN conditions was observed in live yeast-fed pigs. Reasons for the discrepancy between environments are not entirely clear. While dietary yeast has been shown to improve productivity in ruminants (Desnoyers et al., 2009; Moallem et al., 2009), the effects of yeast supplementation in monogastrics are less consistent (Kornegay et al., 1995; Veum et al., 1995; Shen et al., 2017; Kiros et al., 2018). Additionally, previous studies have reported beneficial effects of dietary live yeast (specifically the strain used herein) when supplemented at higher inclusion rates relative to that utilized in the current study (Zanello et al., 2013; Trckova et al., 2014). Therefore, responses to dietary yeast may vary depending on the species, their physiological state, and the type and dose of live yeast supplemented. Furthermore, studies in pigs have primarily focused on the weaning period; thus, evidence regarding live yeast supplementation in growing and finishing pigs, and specifically during HS, is scarce. Future research is warranted to better understand the mechanisms by which dietary live yeast improves overall animal performance, regardless of environmental conditions.

Although yeast supplementation had no effects on any of the blood parameters measured herein, altered post-absorptive metabolism characteristic of HS was observed in the current study. For instance, plasma glucose tended to be decreased and was decreased in HS pigs compared to TN and PF pigs (6% and 10%, respectively). Reduced glucose levels have been previously observed in heat-stressed rats (Mitev et al., 2005), dairy cows (Itoh et al., 1998; Shwartz et al., 2009), and pigs (Sanz Fernandez et al., 2015a, b; Abuajamieh et al., 2018). Decreased circulating glucose during HS may be partially explained by reduced ADFI and the subsequent decreased glucose absorption in the small intestine. A second explanation might be related to increased glucose utilization by the immune system (Kvidera et al., 2017a, 2017b), as we and others have previously reported that HS induces intestinal barrier dysfunction and increases circulating endotoxin (i.e., LPS); consequently, eliciting an inflammatory response (Hall et al., 2001; Pearce et al., 2013b).

Similarly, plasma NEFA levels were decreased in HS relative to both TN and PF pigs, suggesting HS pigs did not mobilize as much adipose tissue as their PF counterparts. This observation agrees with previous studies where animals exposed to HS had decreased NEFA concentrations and increased lipid retention regardless of marked reductions in ADFI (Wheelock et al., 2010; Pearce et al., 2013a; Sanz Fernandez et al., 2015a, b; Baumgard et al., 2011). The lack of lipid mobilization despite pigs being under a catabolic condition like HS seems contradictory, as increased fatty acid oxidation represents a significant source of energy in nutrient-restricted animals (Baumgard and Rhoads, 2013). Reasons why heat-stressed animals fail to make this metabolic “shift” are not clear; however, changes in circulating insulin and thyroid hormones (as discussed below) may partially explain the discrepancy in plasma NEFA levels between HS and PF pigs.

Herein and in agreement with the literature, we observed no changes in circulating insulin between TN and HS pigs regardless of a 30% decrease in ADFI during HS. Additionally, insulin was increased in HS relative to PF pigs, despite both being under the same plane of nutrition. Increased circulating insulin is a hallmark characteristic of HS and has been previously reported in other species, including ruminants and pigs (Wheelock et al., 2010; Pearce et al., 2013a; Sanz Fernandez et al., 2015a, 2015b). Although mechanisms underlying increased insulin during HS remain unclear, the literature suggests insulin plays an important role in activating the cellular stress response (Li et al., 2006). Additionally, insulin secretion might also be influenced by bacterial components (i.e., LPS), as intravenous LPS infusion has been shown to markedly increase insulin levels in both pigs and dairy cows (Kvidera et al., 2017a, 2017b). Furthermore, we and others have demonstrated that HS reduces intestinal integrity and increases circulating LPS (Lambert et al., 2002; Pearce et al., 2013b); therefore, immune activation-induced insulin secretion during HS may represent a strategy to divert nutrients to the immune system to support an inflammatory response (as reviewed by Baumgard et al., 2016).

In the current study, plasma T_3_ and T_4_ were decreased in HS when compared to TN and PF pigs. These results are similar to those previously reported where thyroid hormones were markedly reduced during a heat load (Prunier et al., 1997; Johnson et al., 2015; Sanz Fernandez et al., 2015a). As mentioned before, thyroid hormones are key regulators of energy metabolism (Kim, 2008); therefore, reduced circulating levels of both T_3_ and T_4_ suggest a decrease in basal metabolic rate and heat production and may partially explain why maintenance costs are, in fact, decreased during HS. Furthermore, changes observed in lipid metabolism may also result from variations in circulating thyroid hormones. Considering that both T_3_ and T_4_ stimulate lipolysis and fatty acid utilization by extrahepatic tissues (Pucci et al., 2000; Sinha et al., 2018), their decrease in circulation may partially contribute to the absence of adipose tissue mobilization observed in the current study.

As previously stated, reduced intestinal integrity is a common observation in animals exposed to an excessive thermal load (Lambert et al., 2002; Pearce et al., 2013b). Increased intestinal permeability and the subsequent LPS translocation across the gut barrier trigger an inflammatory response that is usually accompanied by increased production of circulating pro-inflammatory cytokines, like TNF-α (Lambert et al., 2002). Interestingly, no changes in circulating TNF-α were observed in pigs exposed to HS in the current study. Although surprising, this observation agrees with previous trials where pigs exhibited a reduced cytokine response when exposed to a thermal load (Pearce et al., 2015; Abuajamieh et al., 2018; Gabler et al., 2018; Mayorga et al., 2019). Reasons for the decreased inflammatory response are unclear but likely involve HS-induced activation of heat shock proteins and their role in inhibiting the expression of the nuclear factor kappa B (**NF-kB**) pathway, which regulates the inflammatory response (Schell et al., 2005; Janus et al., 2011). Conversely, HS-related adaptations in liver function might play a role wherein the efficiency of clearance of gut-derived endotoxin might be increased (Fox et al., 1989), though investigating this premise was beyond the scope of the present study. Interestingly, despite no differences observed during HS, a post hoc analysis revealed that circulating TNF-α tended to be decreased and was numerically decreased in HS and PF pigs fed live yeast, respectively, compared to their Con counterparts. Reasons for decreased TNF-α during both HS and nutrient restriction are not entirely clear; however, the immunomodulatory effect of yeast has been mainly attributed to the presence of β-_D_-glucans and α-_D_-mannans in its cell wall (Broadway et al., 2015). While β-_D_-glucans are believed to prime the immune system by altering the cytokine response and increasing antibody production, α-_D_-mannans are thought to prevent bacterial adhesion to the intestinal lumen, thus protecting the gut against colonization by enteric pathogens (Kogan and Kocher, 2007). Regardless, understanding why live yeast-fed pigs had decreased cytokine levels and whether this observation is biologically relevant remains to be elucidated.

In conclusion, chronic HS exposure increased all body temperature indices and decreased production parameters (i.e., ADFI, ADG, and final BW) but did not affect G:F. Furthermore, pigs exposed to HS exhibited altered post-absorptive metabolism as indicated by decreased circulating glucose, reduced NEFA, increased insulin, and reduced thyroid hormones. Live yeast supplementation tended to improve G:F under TN conditions. However, dietary live yeast did not affect body temperature indices, growth performance, or circulating markers of metabolism during HS or nutrient restriction, but it tended to decrease circulating TNF-α in HS pigs. It is not clear what the practical implications of reduced TNF-α are, but presumably, a reduction in the inflammatory tone is a positive result. Consequently, further research is needed to better understand the effects of dietary live yeast in pigs during HS or nutrient restriction scenarios.
